# Overexpression of MiR482c in Tomato Induces Enhanced Susceptibility to Late Blight

**DOI:** 10.3390/cells8080822

**Published:** 2019-08-03

**Authors:** Yu-Hui Hong, Jun Meng, Xiao-Li He, Yuan-Yuan Zhang, Yu-Shi Luan

**Affiliations:** 1 School of Bioengineering, Dalian University of Technology, Dalian 116024, China; 2 School of Computer Science and Technology, Dalian University of Technology, Dalian 116024, China

**Keywords:** tomato, late blight, miRNA, overexpression, resistance

## Abstract

Tomato is the highest-value fruit/vegetable crop worldwide. However, the quality and yield of tomatoes are severely affected by late blight. MicroRNA482s (miR482s) are involved in the plant’s immune system. In this study, miR482c was transiently and stably overexpressed in tomatoes in transgenic plants to explore its mechanism in tomato resistance against late blight. Transgenic tomato plants with transiently overexpressed miR482c displayed a larger lesion area than the control plants upon infection. Furthermore, compared with wild-type (WT) tomato plants, the transgenic tomato plants stably overexpressing miR482c displayed a decreased expression of target genes accompanied by lower peroxidase (POD), superoxide dismutase (SOD), and phenylalanine ammonia-lyase (PAL) activity activities and higher malondialdehyde (MDA) content, thereby leading to a decline in reactive oxygen species (ROS) scavenging ability and aggravating the damage of lipid peroxidation product accumulation on the cell membrane, eventually enhancing plant susceptibility. This finding indicates that miR482c may act as a negative regulator in tomato resistance by regulating nucleotide binding sites and leucine-rich repeat (NBS-LRR) expression levels and ROS levels.

## 1. Introduction

Tomato (*Solanum lycopersicum*) is the highest-value fruit/vegetable crop worldwide with considerable significance for human nutrition and health [1,2]. In the last five years, the average annual production of tomatoes was nearly 96 million tons according to data from the Food and Agriculture Organization of the United Nations (FAOSTAT) [3]. The yield and quality of tomatoes are seriously affected by late blight in both pre- and postharvest stages [4]. It has been shown that an unprotected tomato crop (greenhouse, field) can be devastated within seven to ten days once infected with late blight [5]. The management of late blight is relatively difficult, as this disease destroys foliage and fruits throughout the whole growth and development period of tomato [5,6]. Traditional strategies for plant resistance rely on the frequent use of antimicrobials, leading to serious pollution of the environment. To date, genetically engineering resistant cultivars has become the most efficient and economical long-term approach to strengthening plant resistance to various forms of stress. Due to a long breeding cycle and rapid pathogen variation, it is difficult for traditional disease-resistant breeding approaches to meet the needs of agricultural production. Comprehending the molecular mechanism of plant–pathogen interactions and exploring disease resistance genes are key to enhancing plant resistance.

MicroRNAs (miRNAs), as a class of 22–24 nucleotide (nt) noncoding RNAs, play significant roles in most eukaryotes by regulating the posttranscription of target genes [7,8]. MiRNAs have been shown to bind target mRNA molecules at complementary sites and thereby trigger target degradation or translational inhibition [9]. In recent years, mounting studies have reported that miRNA cascades influence plant response to biotic stress [10]. For instance, using small RNA sequencing, Luan et al [11] identified 70 significantly changed miRNAs in tomato in response to late blight. MiR393 in Arabidopsis was shown to enhance plant immune systems and contribute to plant defense against bacteria [12]. MiR164a in rice was downregulated, and its target transcription factor OsNAC60 was derepressed correspondingly to enhance plant defenses against rice blast [13]. MiR1916 in tomato was demonstrated to negatively regulate tomato resistance to late blight and gray mold [14]. In addition to acting as regulators under biotic stress, miRNAs also participate in the response to abiotic stress and plant development. For example, miR169c contributes to tomato tolerance to drought by regulating targeted three nuclear factor Y subunit genes and one resistance-associated gene [15]. MiR171b participates in organ development by downregulating several GRAS genes [16]. The direct binding of ripening inhibitors (RIN) to the promoter of miR172a in tomato suggests that, in fruit development and ripening, there is a close correlation between RIN and miRNA expression [17].

Conserved miRNAs mostly exist as gene families in the genome, and more than 90% of the conserved miRNA gene families are multigene families [18]. The conservation of different member sequences in the plant miRNA family suggests a similar or synergistic function. For instance, activating miR166k-166h expression in rice enhanced plant resistance to rice blast and bakanae disease [19]. Tomato plants overexpressing miR172a and miR172b exhibited enhanced resistance to late blight by suppressing an AP2/ERF transcription factor [20]. Silencing miR2118b (corresponding to sly-miR482d in the miRBase) and miR482e conferred resistance to late blight to tomato [21]. In addition, several studies have shown that members of the same gene family may play different roles in the immune system of plants. For example, Csukasi et al. [22] documented that during the course of strawberry receptacle development, treatment with gibberellin downregulated miR159a expression but that miR159b expression remained unchanged. Ma et al. [23] found via small RNA sequencing that miR156b and miR156e were significantly induced in two peanut eighth-generation recombinant inbred lines (RIL8) at 35 days after flowering, whereas the expression of miR156o was significantly reduced. Moreover, in rice, the overexpression of miR164c led to reduced germination rates [24], while miR164b negatively regulated drought resistance by cleaving NAC genes [25]. These studies suggested that members of miRNA families may act differently in response to diverse stresses in various plant species.

MiR482 is an ancient and extensive family present in all land plants which performs a regulatory function by cleaving and inhibiting the expression of target nucleotide binding sites and leucine-rich repeat (NBS-LRR) mRNAs [26]. NBS-LRR, as a class of Resistance (R) genes, recognize effectors in a direct or indirect way and can redirect the defense signaling and trigger R-gene-mediated immunity [27]. The miR482 family is more labile in sequence than other miRNA families [26,27,28]. To some extent, its diversity is a result of the amino acid variation in its target sequence [29]. Additionally, the expression of miR482 in the Solanaceae species is higher than that in other species [26]. The miR482 family in *Solanum lycopersicum* possesses five members, namely, sly-miR482a/b/c/d/e [30,31,32]. The length of all five members is 22 nt rather than the typical 21 nt [26]. As members of the miR482 family in tomato display low sequence conservation with sequence variation at 5/6 variable sites [21], we hypothesize that the functions of members may differ. A recent study showed that sly-miR482e and sly-miR2118b (corresponding to sly-miR482d in the miRBase) differed in structure and function, which resulted in differences in their targets and mechanisms [21]. In our previous study, silencing sly-miR482b with a short tandem target mimic (STTM) was able to confer enhanced resistance to late blight in transgenic tomato [33]. The sequence alignment of sly-miR482b and sly-miR482c showed that their sequences were identical at 19 sites and varied at three sites. This difference indicated the possibility that miR482b and miR482c target distinct genes and function in different ways. Here, we overexpressed miR482c in tomato to study its mechanism. Two NBS-LRR genes with coiled-coil domains (CNLs), *Solyc07g049700* and *Solyc11g006530,* were predicted to be targeted by miR482c according to previous studies [26,34]. The co-regulation of miR482c and these two genes was identified in tomato (*S. lycopersicum* cv. Heinz 1706) resistant to *Phytophthora infestans* IPO-C [34]. The genes *Solyc07g049700* and *Solyc11g006530* were selected and named *SlCNL1* and *SlCNL2,* respectively. Here, our study complemented the previous work and further explained the relationship and difference between miR482c and miR482b, laying a solid foundation for the continued work.

## 2. Materials and Methods

### 2.1. Plant Strains, Phytophthora Infestans Infections

Tomato Zaofen No. 2 (susceptible cultivar) plants were raised in a growth room with a 16 h light (at 22 °C) and 8 h dark (at 18 °C) period. The agent of late blight, *Phytophthora infestans* (*P. infestans*) “P12103”, was cultured in oat medium at 20 °C without light for 20 days, and the plates covered with mycelium were rinsed with distilled water until the concentration of spores reached 10^6^ zoospores·mL^−1^. Afterwards, the spore suspensions were placed at 4 °C for 1–2 h to release the zoospores. Tomato plants with six fully expanded true leaves were sprayed with the prepared spore suspension. The control group consisted of tomato plants sprayed with sterile water. Each group consisted of three seedlings. Leaf samples were collected at 0, 6, 24, 48, 72, and 96 h post inoculation (hpi) according to a prior study [34]. All samples were quickly frozen in liquid nitrogen and subsequently stored at −80 °C until DNA and RNA extraction.

### 2.2. Construction of the MiR482c-Overexpression Plasmids

The precursor sequence of miR482c (miRBase Accession: MI0020250) was amplified from tomato genomic DNA. The gene-specific primer pairs (listed in Table S1) were designed by Oligo version 7.6 software. The PCR products were cloned into the pBI121 vector driven by the 35S promoter. The sequence was identified by DNA sequencing.

### 2.3. Transient Overexpression of MiR482c in Tomato

The plasmids pBI121-miR482c were introduced into the *Agrobacterium tumefaciens* (*A. tumefaciens*) strain “GV3101” by the freeze-thaw method [35]. The culture of *A. tumefaciens* was centrifuged for 10 min at 4000× *g*, and the pellet was resuspended in infiltration medium (10 mM MES, 10 mM MgCl_2_, and 20 µM acetosyringone; OD_600_ = 1.0). Next, the *A. tumefaciens* cultures containing pBI121-miR482c were agroinoculated into the leaves of the tomato plants. The infiltration with *A. tumefaciens* cultures containing empty vectors was used as the control. Three days after infiltration, each infiltrated leaf was inoculated with 20 µL of *P. infestans* spore suspension (10^6^ zoospores·mL^−1^) at the same position. The necrotic areas were observed after 3 days. Statistical differences in the diameters of necrotic lesions (DOL) between the control and infections were estimated using Duncan multiple range tests.

### 2.4. Generation of Transgenic Tomato Overexpressing MiR482c

The transformation of tomato was performed via the Agrobacterium-mediated leaf disk method [36]. Explants were obtained from cotyledons excised from one-week-old seedlings. The putative resistant buds were selected on 1/2-strength Murashige and Skoog medium containing 50 mg·L^−1^ kanamycin and 200 mg·L^−1^ carbenicillin with a 16 h light (at 22 °C) and 8 h dark (at 18 °C) period. After nearly two months, the putative positive transgenic lines were further confirmed by PCR amplification of the kanamycin-resistant gene neomycin phosphoryl transferase II (*nptII*) (listed in Table S1). The expression levels of miR482c in the selected transgenic lines were determined using quantitative real-time PCR (qRT-PCR).

### 2.5. Disease Resistance of Transgenic Plants Overexpressing MiR482c

The transgenic plants and wild-type (WT) plants with consistent growth were selected for the evaluation of resistance. In the detached-leaf infection assay, three leaves per plant and three plants per line were detached from one-month-old plants and placed on filter paper in a glass garden. A pipette tip (0.5–10 µL) was used to gently stab a wound to the right of the midvein on the abaxial side of the leaf. Next, 20 μL spore suspensions of *P. infestans* were injected into the wound. The plates were incubated in the dark at 20 °C. The disease symptoms on inoculated leaves were observed and measured at 5 days postinfection. Subsequently, the leaves were dyed in trypan blue solution (2.5 mg·mL^−1^) at 23 ± 1 °C for 12 h and discolored by being soaked in lactophenol/ethanol solution [14].

In the whole-plant infection assay, the WT plants and transgenic plants were sprayed with the same zoospore suspension and maintained in the greenhouse without light at 20 ± 1 °C. The humidity was kept at 95%. After one day, a 16 h light and 8 h dark period was provided. Five days after infection, the disease severity rating (DSR) [37] was counted. Additionally, the abundance of miR482c and its target genes in the WT and transgenic plants were detected using qRT-PCR.

### 2.6. DAB/NBT Staining and Measurements of Hydrogen Peroxide/MDA Content

At 5 days postinfection, the transgenic plants and WT plants with consistent growth were selected for analysis of H_2_O_2_/O_2_^−^ with diamino benzidine (DAB)/nitro blue tetrazolium (NBT) staining as reported by previous methods [38]. Moreover, the activities of peroxidase (POD) and superoxide dismutase (SOD) were measured according to a previous study [35]. Furthermore, the content of malondialdehyde (MDA) and the activity of phenylalanine ammonia-lyase (PAL) were determined according to methods described by Hong et al. [37].

### 2.7. qRT-PCR Analysis

The relative expression levels of all genes in this work were determined by qRT-PCR. The synthesis of cDNA and qPCR of miRNA were performed following the protocol from the *TransScript®* Green miRNA Two-Step RT-PCR SuperMix kit (TransGen Biotech, Beijing, China). A poly (A) tail was added to the 3’ end of the miRNA by poly (A) polymerase. Reverse transcription was carried out to generate cDNA by using a universal primer containing oligo (dT) at the 5’ end. Relative expression of miRNAs was detected by PCR under the action of *TransStart®* Tip Green qPCR SuperMix, according to the manufacturer’s instructions. The universal primer containing oligo (dT) at the 5’ end was supplied in kit and used for the reverse transcription as well as miRNA qPCR. The primers for candidate reference and target genes are listed in Table S1. The tomato housekeeping gene *actin* was selected as the reference gene [39]. All reactions were repeated three times. Together, three independent biological assays (three seedlings each assay) were performed. The relative expression levels of each gene were calculated using the 2^−ΔΔCT^ method.

### 2.8. Statistical Analysis

Statistical differences in data were estimated by Duncan multiple range tests using IBM SPSS Statistics19 software. Significant differences (*p* < 0.05) are presented with different lowercase letters. All data are presented as the means ± standard deviations (SDs).

## 3. Results

### 3.1. Expression Patterns of MiR482c and Its Target Genes upon Infection

**Figure 1:**
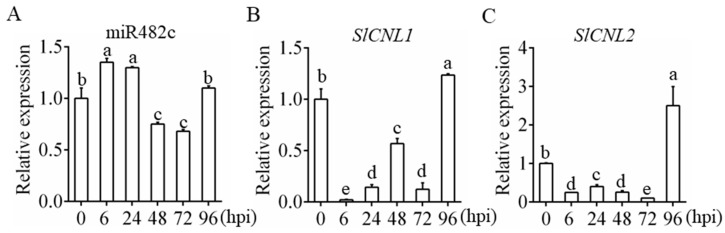
MiR482c and *SlCNL* expression patterns upon infection. (**A**,**B**,**C**) The abundance of miR482c (**A**), *SLCNL1* (**B**), and *SlCNL2* (**C**) in wild-type (WT) plants upon infection. hpi: hours postinfection. The tomato housekeeping gene *actin* was selected as the reference gene. standard deviations (SDs) of three replicates are indicated with error bars. The letters above the column indicate a significant difference (*p* < 0.05) between the samples.

The relative expression levels of miR482c and *SlCNL*s upon infection are shown in Figure 1. The relative expression of miR482c was significantly upregulated at 6 hpi, subsequently downregulated at 48 hpi, and eventually upregulated at 96 hpi (Figure 1A). Moreover, the expression patterns of *SLCNL1* (Figure 1B) and *SLCNL2* (Figure 1C) was significantly downregulated at 6 hpi, then upregulated at 24 hpi, showing contrasting trends to that of miR482c. After 48 hpi, the expression pattern of *SLCNL1* and *SLCNL2* were similar to that of miR482c. It is speculated that miR482c and *SLCNL*s were induced early in infection, and there is a targeted relationship between miR482c and *SLCNL*s.

### 3.2. Transient Overexpression of MiR482c Compromised Tomato Resistance

**Figure 2:**
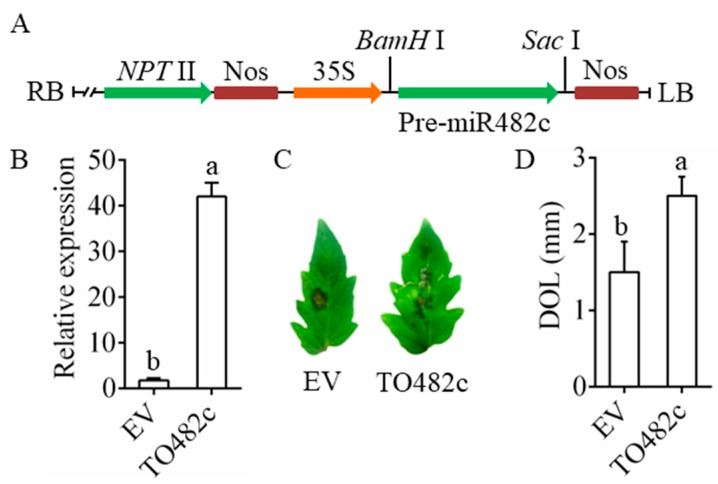
Transient overexpression of miR482c in tomato leaves. (**A**) The framework of the miR482c-overexpression vectors. (**B**) The expression level of miR482c in tomato that transiently overexpressed miR482c. EV: empty vector; TO482c: transiently overexpressing pBI121-miR482c. (**C**) The necrotic lesions on the leaves of EV and TO482c plants on the 3rd day after inoculation. (**D**) The diameters of necrotic lesions (DOL) in EV and TO482c plants. SDs of three replicates are indicated with error bars. The letters above the column indicate a significant difference (*p* < 0.05) between the samples.

The framework of the pBI121-miR482c overexpression plasmids is shown in Figure 2A. The control transiently overexpressed empty vector (EV) in tomato leaves. The abundance of miR482c in tomato transiently overexpressing miR482c (TO482c) was nearly 20-fold higher than that in the control (Figure 2B). Compared with the control plants, the TO482c plants displayed more necrotic areas at 3 days after leaf infiltration (Figure 2C). In addition, the DOL on the TO482c leaves were larger than those on the control leaves (Figure 2D). The above results indicated that transiently overexpressing miR482c might compromise tomato resistance.

### 3.3. MiR482c Had a Negative Effect on Tomato Resistance

**Figure 3:**
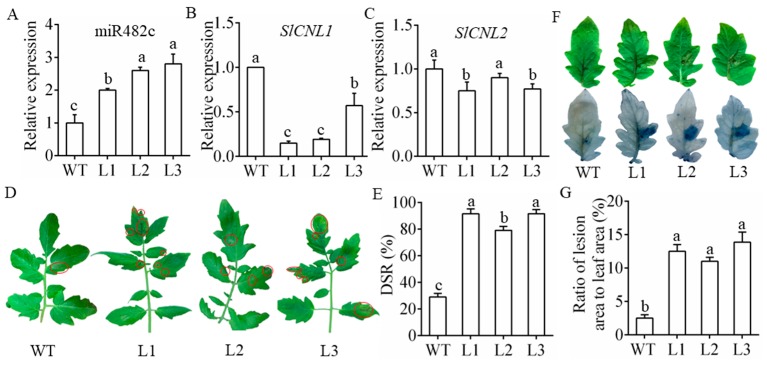
Disease resistance of transgenic plants stably overexpressing miR482c. (**A**, **B**, **C**) The relative expression levels of miR482c (**A**), *SlCNL1* (**B**), and *SlCNL2* (**C**) in transgenic tomato lines. WT: wide type; L1: line 1; L2: line 2; L3: line 3. (**D**) The disease symptoms of WT and L1–L3 in the whole-plant infection assay on the 5th day after infection. (**E**) The disease severity rating (DSR) of WT and L1–L3 on the 5th day after infection. (**F**) Necrotic areas on detached leaves of the WT and L1–L3 plants on the 5th day after inoculation. Top: necrotic areas; bottom: trypan blue staining of the detached leaves. (**G**) The ratio of lesion area to leaf area in the detached-leaf infection assay. SDs of three replicates were displayed by error bars. The letters above the column indicate a significant difference (*p* < 0.05) between the samples.

The pBI121-miR482c plasmids were introduced into tomato to generate transgenic plants to further determine the regulation of miR482c in the resistance of tomato to late blight. Three positive transgenic lines (L1, L2, and L3) were selected for further examination. The expression patterns of miR482c and *SlCNL*s in the transgenic lines were determined by qRT-PCR. As shown in Figure 3A, miR482c was more abundant in the three transgenic lines than in the WT plants, with the expression levels of the three transgenic lines L1, L2, and L3 ~2-fold, 2.5-fold, and 2.8-fold higher, respectively, than those of WT. Moreover, the transcript levels of *SlCNLs* in the miR482c-overexpressing plants were lower than those in the WT plants (Figure 3B,C). The decline in *SlCNL* mRNA levels in the transgenic plants further demonstrated that *SlCNL* mRNAs were targeted by miR482c.

In the whole-plant infection assay, more severe disease symptoms (indicated with the red circles) were observed on the leaves of the L1–L3 plants than on the leaves of the WT plants (Figure 3D). The DSR of the L1–L3 plants was almost 4-fold higher than that of the WT plants (Figure 3E). In the detached-leaf inoculation assay, dead cells were detected in the leaves stained with trypan blue after infection. The L1–L3 leaves had darker blue marks than did the WT leaves, indicating increased cell death in the L1–L3 plants (Figure 3F). Additionally, the ratio of lesion area to leaf area in the leaves of the WT plants was much less than that of the leaves of the transgenic tomato plants (Figure 3G).

### 3.4. Overexpression of MiR482c Contributed to Changes in Physiological Indicators

**Figure 4:**
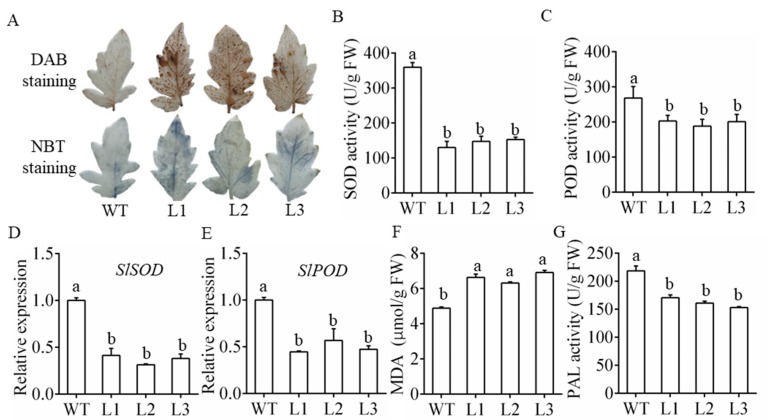
Detection of physiological and biochemical indicators of the miR482c-overexpression plants. (**A**) Diamino benzidine (DAB) staining and nitro blue tetrazolium (NBT) staining of tomato leaves in the whole-plant infection assay on the 5th day after infection. (**B**,**C**) SOD (**B**) and POD (**C**) activity of WT and L1–L3 after infection for 5 days. (**D**,**E**) The expression levels of *SlSOD* (**D**) and *SlPOD* (**E**) in WT and transgenic lines on the 5th day after infection. (**F**) The malondialdehyde (MDA) content of WT and L1–L3 after infection for 5 days. (**G**) The phenylalanine ammonia-lyase (PAL) activity of WT and L1–L3 after infection for 5 days. SDs of three replicates are indicated with error bars. The letters above the column indicate a significant difference (*p* < 0.05) between the samples.

In plant-pathogen interactions, oxidative bursts frequently occur and produce large amounts of reactive oxygen species (ROS), mainly including H_2_O_2_ and O_2_^−^. In this study, DAB staining and NBT staining were conducted to determine the accumulation of H_2_O_2_ and O_2_^−^ in the transgenic plants and WT plants after treatment. As shown in Figure 4A, the leaves of the transgenic plants exhibited more intensely stained spots than those of WT. Additionally, the enzyme activities of POD and SOD were significantly lower in the L1–L3 plants than in the WT plants upon infection (Figure 4B,C). This was consistent with the notably decreased expression of *SlSOD* and *SlPOD* (genes encoding the SOD and POD enzymes in tomato) [20,35] in the L1–L3 plants compared with the WT plants upon infection (Figure 4D,E). These results indicated that the L1–L3 plants accumulated more ROS than the WT plants.

In addition, a higher MDA content was found in the L1–L3 plants, indicating that the L1–L3 plants suffered more severe membrane damage than the WT plants (Figure 4F). PAL is a key enzyme in the phenylpropanoid metabolic pathway. The level of PAL activity can also be used as an important physiological indicator for measuring plant disease resistance. The activity of the PAL enzyme in the L1–L3 plants was higher than that in the WT plants (Figure 4G). These results suggest that overexpressing miR482c in tomato aggravates membrane damage.

## 4. Discussion

MiRNAs, as key regulators, have been widely suggested to play crucial roles in plant resistance. A large number of studies have documented that miRNAs function in plant response to biotic stress. For instance, osa-miR167d has been reported to act as a negative regulator in rice resistance to rice blast disease [40]. Osa-miR396 has been demonstrated to negatively regulate the resistance of rice to brown planthopper (BPH) [41]. In Arabidopsis, miR825 and miR825* were shown to compromise plant resistance to *Pseudomonas syringae* pv. *tomato* (*Pst*) DC3000 [42]. In addition to acting as regulators under biotic stress, miRNAs also participate in the response to abiotic stress. For example, miR268 functions in tolerance to Cd stress by cleaving resistance-associated proteins [43]. miR2118/miR482 was upregulated under drought stress [44,45]. miR482 was downregulated under ABA and NaCl stress [46]. Tomato plants are generally subject to various pathogens leading to severe diseases and substantial losses. However, the mechanism of miR482c in tomato disease resistance remains elusive. Our study indicates that overexpression of miR482c in tomato leads to enhanced susceptibility to late blight disease.

We found that the expression pattern of miR482c was induced at 6 hpi and 24 hpi, subsequently declined at 48 hpi and 72 hpi, and finally increased at 96 hpi (Figure 1A). The relative expression levels of both *SLCNL1* and *SLCNL2* decreased at 6 hpi and were subsequently upregulated at 24 hpi in contrast. Afterwards, at 48 hpi, *SLCNL1* increased, whereas *SLCNL2* decreased. Both *SLCNL*s declined at 72 hpi and were eventually upregulated at 96 hpi (Figure 1B,C). A previous study demonstrated that the co-regulations between miR482-NBS-LRRs were not exactly the same [34]. Such differences in co-regulation suggest that, despite active targeting by miR482c in *S. lycopersicum*, *SlCNL1* and *SlCNL2* are likely to be regulated by other mechanisms in addition to the regulation by miR482c. Furthermore, upregulation or downregulation is not static between miR482 and NBS-LRR mRNA but can shift between time points. Switches between translational and posttranscriptional may account for such shifted regulation [34]. Additionally, Jiang et al. demonstrated that the abundance of miR482b in L3708 tomato (resistant cultivar) was lowest at 24 hpi but increased to a moderate level at 96 hpi [33]. The different expression patterns of mi482b and miR482c may result from divergent cultivars.

To further confirm the participation of miR482c in tomato resistance, three transgenic lines overexpressing miR482c (L1–L3) were generated. After five days postinfection, there was a clear downregulation of *SlCNL1* in transgenic plants L1, L2 and L3 yet this was not so clear for *SlCNL2* (Figure 3C). Such differences may suggest that despite active targeting by miR482c in *S. lycopersicum*, *SlCNL1* and *SlCNL2* are likely to be regulated by other mechanisms in addition to the regulation by miR482c. Despite being targeted by sly-miR482c in our work, *SlCNL2* was also targeted by sly-miR482b (miRBase Accession: MI0020250) according to a previous report [26]. The complex and diversified regulatory network between miRNAs and their target genes may account for this phenomenon. After five days postinfection, the transgenic lines displayed more severe symptoms than the WT (Figure 3D) with greater DSRs (Figure 3E). Moreover, in the detached-leaf inoculation assay, necrotic areas on the leaves of transgenic lines were larger than those on the leaves of the WT (Figure 3F). This indicated that the resistance was impaired after overexpression of miR482c. Previous work reported similar results; the mature sequences of stu-miR482e in potato and sly-miR482c in tomato are identical, and there are only two site variations between the precursor sequences of stu-miR482e and sly-miR482c. Overexpression of stu-miR482e in potato was demonstrated to enhance plant sensitivity to *Verticillium dahliae* infection [47].

In the early stages of plant–pathogen interactions, oxidative bursts frequently occur and produce large amounts of ROS, mainly including H_2_O_2_ and O_2_^−^ [48]. Excessive ROS may result in lipid peroxidation and oxidative damage to the membrane or even cell death. In rice, Wu et al. showed that miR528 acts as a negative regulator in viral resistance and is accompanied by an increased accumulation of ROS [49]. In our study, the DAB and NBT staining assay showed that there were more dark spots on the leaves of the transgenic tomato plants than the leaves of the WT tomato plants, indicating increased cell death and accumulation of H_2_O_2_ and O_2_^−^ in the transgenic tomato plants (Figure 4A). Plants have evolved a complete antioxidant defense system that removes excess ROS and maintains homeostasis. The activities of SOD and POD related to ROS clearance in the transgenic tomato plants were considerably lower upon infection than those in the WT plants (Figure 4B,C). Together, the relative expression levels of the antioxidant enzyme genes *SlPOD* and *SlSOD* were also downregulated in the transgenic plants (Figure 4D,E). Similar results have been reported in tomato, for example, Zhang et al. reported that *slmapk3* mutants and WT showed increased accumulation of ROS upon *Botrytis cinerea* infection, and *slmapk3* mutants showed reduced resistance with an increased level of ROS content [50].

**Figure 5:**
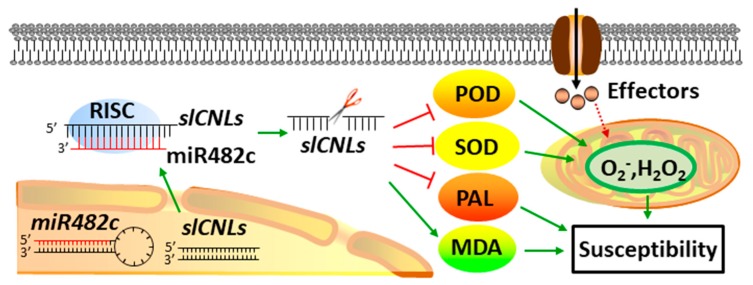
The regulation network of miR482c- nucleotide binding sites and leucine-rich repeat (NBS-LRR) involved in tomato resistance. Overexpressing miR482c in tomato enhanced plant susceptibility to late blight by reducing the targeted *SlCNL* genes, accompanied by lower POD, SOD, and PAL activities and higher MDA content. Red lines represent negative regulated gene or function, while green arrows represent positive regulated gene or function. Red dotted arrow indicates the effectors produced by pathogens invading the host plant acts on the production of ROS by a direct or indirect way.

In summary, we proposed a working model to describe the mechanism of miR482c in tomato resistance to late blight (Figure 5). Overexpression of miR482c could reduce the expression of its targeted *SLCNL* genes and antioxidant enzyme genes, thereby leading to a decline in the ROS scavenging ability and aggravating the damage of lipid peroxidation product accumulation on the cell membrane, eventually enhancing plant susceptibility. Compared with the function of miR482b which was reported earlier [33], miR482c showed analogous negative regulation with miR482b in tomato resistance to late blight. Thus, despite having individual characteristics, miR482c and miR482b may play analogous roles in the plant immune system. Results have been reported that coincide with this finding. For instance, Li et al. reported that miR169a and miR169c in Arabidopsis functioned as negative regulators under drought stress by cleaving NFYA5 [51]. Furthermore, Zhao et al. demonstrated that miR169g and miR169n both targeted an NF-YA gene exhibiting distinct and overlapping responses to salt and drought stresses [52].

Our findings provide insight into the method by which miR482c-NBS-LRR participated in the response of tomato to late blight. Future work should therefore include follow-up work designed to study whether silencing miR482c enhances disease resistance and whether the miR482c cascade plays a role in resistance to other forms of stress. 

## Supplementary Materials

The following are available online at https://www.mdpi.com/2073-4409/8/8/822/s1, Table S1: Primers used for this study.

## Abbreviations Used

H_2_O_2_	hydrogen peroxide
MDA	malondialdehyde
NBT	malondialdehyde
O_2_^−^	superoxide
PAL	phenylalanine ammonia-lyase
POD	peroxidase
qRT-PCR	quantitative real-time PCR
ROS	reactive oxygen species
SD	standard deviation
SOD	superoxide dismutase
WT	wild type
