# Supplementary material for: Overexpression of MiR482c in Tomato Induces Enhanced Susceptibility to Late Blight
Source: Cells. 2019 Aug 3;8(8):822. doi: 10.3390/cells8080822 (PMC6721620; doi:10.3390/cells8080822)
Supplement: Supplementary file 1 [file cells-08-00822-s001.pdf]

Table S1 Primers used for this study.

**Purpose**	**Gene Name**	**Primer Sequence (5' - 3')**
clone of pre-miR482c	c-miR482c	FP:CGAGCTCCCCAGCTTTCATCATGTTTTTCA
		RP:CGGGATCCGAAGCCATTGGAGTTAAGTTGACA
identification of transgenic lines	*nptII*	FP: CTCACCTTGCTCCTGCCGAGA
		RP: CGCCTTGAGCCTGGCGAACAG
qRT-PCR	miR482c	FP:TCTTGCCAATACCGCCCATTCC
	tomato *actin*	FP:ACCTTCAACGTTCCAGCTATG
		RP:TCACCAGAGTCCAACACAATAC
	SLCNL1	FP:TGGATGCACATGAAATCGGC
		RP:TCCAAATCTCCGTTGGGAGC
	SLCNL2	FP:TGTTGGGATGGAGGATGACTT
		RP:TCTTACCTATACCGCCCATTCC
	SlPOD	FP: GAATGTCCGGGAGTTGTTTCT
		RP: TCTTCTACCAGTTGGCACATTC
	SlSOD	FP: AGAAAGCTGTTGCTGTCCTTA
		RP: CCAGGAGCAAGTCCAGTTATAC
